# Supplementary material for: Ouabain Counteracts Retinal Ganglion Cell Death Through Modulation of BDNF and IL-1 Signaling Pathways
Source: Brain Sci. 2025 Jan 26;15(2):123. doi: 10.3390/brainsci15020123 (PMC11853102; doi:10.3390/brainsci15020123)
Supplement: Supplementary file 1 [file brainsci-15-00123-s001.zip › brainsci-3410536-supplementary.pdf]

Supplementary Material

Ouabain counteracts retinal ganglion cell death through modulation of BDNF and IL-1 signaling pathways

Amanda Candida da Rocha Oliveira[1,2], Camila Saggioro de Figueiredo[1,2], Ícaro Raony[3], Juliana Salles Von-Held-Ventura[1], Marcelo Gomes Granja[1,4], Thalita Mázala-de-Oliveira[1,2], Vinicius Henrique Pedrosa Soares[1], Aline Araujo dos Santos[2,5], Elizabeth Giestal-de-Araujo[1,2*]

[1] Department of Neurobiology and Program of Neurosciences, Institute of Biology, Federal Fluminense University, Niteroi, Brazil;

[2] National Institute of Science and Technology on Neuroimmunomodulation - INCT-NIM, Oswaldo Cruz Institute, Oswaldo Cruz Foundation, Rio de Janeiro, Brazil;

[3] Institute of Medical Biochemistry Leopoldo de Meis, Federal University of Rio de Janeiro, Rio de Janeiro, Brazil;

[4] Research, Innovation, and Surveillance Center for Covid-19 and Health Emergencies, Oswaldo Cruz Foundation, Rio de Janeiro, RJ, Brazil;

[5] Department of Physiology and Pharmacology and Program of Neurosciences, Biomedical Institute, Federal Fluminense University, Niteroi, Brazil;

* Correspondence: egiestal@id.uff.br

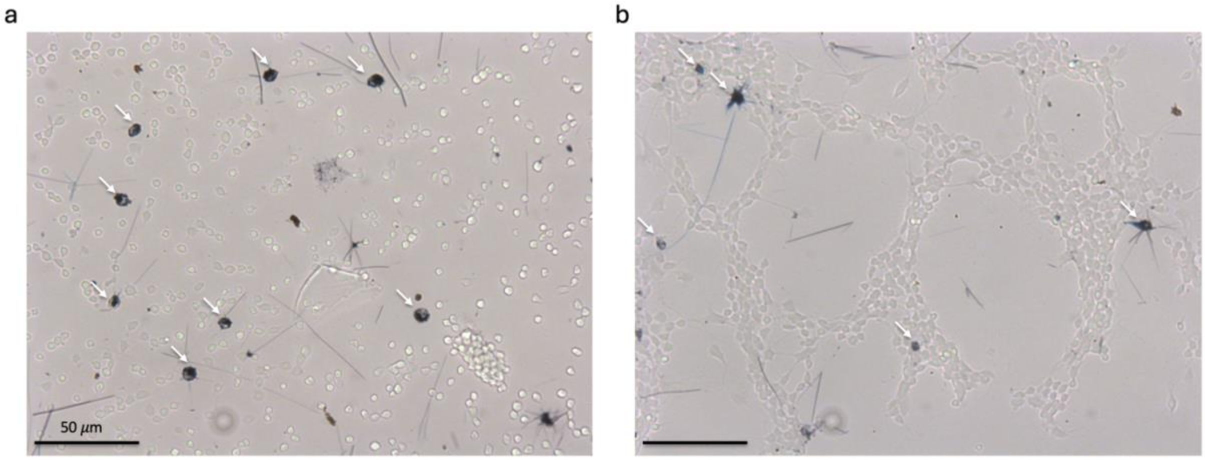

Figure S1. Retrograde labelling of RGCs. Representative images of RGCs labelled with HRP in untreated retinal cell cultures after 4 h (a) and 48 h (b). Scale bars: 50 µm; arrows show HRP-labeled RGCs.

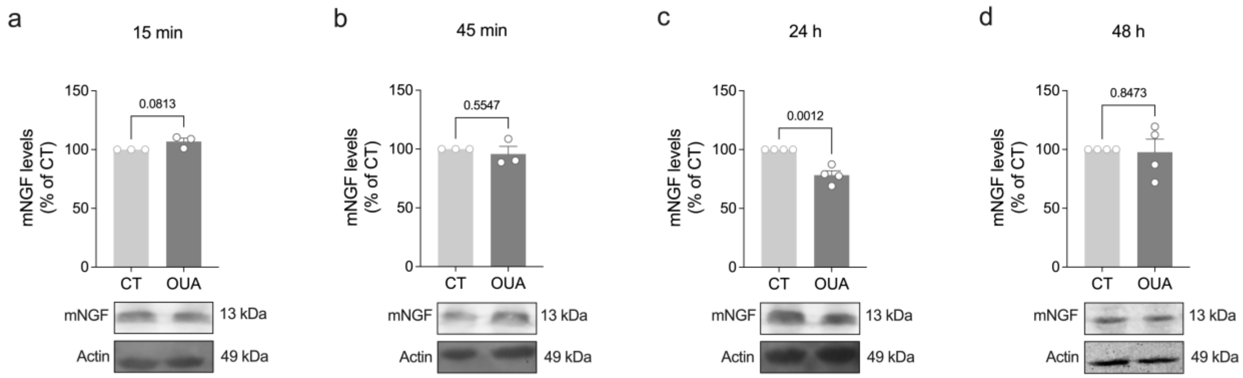

Figure S2. Levels of mature NGF in retinal cells after exposure to ouabain. (**a-d**) Primary retinal cultures were treated with ouabain (OUA; 3 nM) for the indicated periods to assess levels of mNGF by Western blot. Plots represent mean (±SEM) protein optical density (normalized to actin) expressed as a percentage of control (CT) group (set on 100%), with N = 3-4 experiments using independent retinal cultures. Data were analyzed by two-tailed Student t-test.

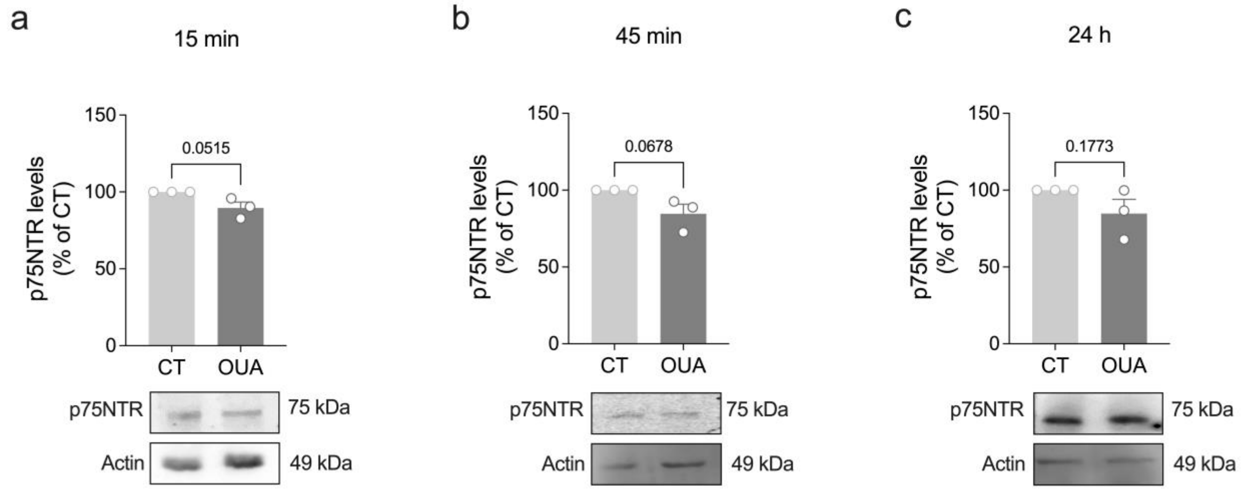

Figure S3. Levels of p75 neurotrophin receptor in retinal cells after exposure to ouabain. (**a-c**) Primary retinal cultures were treated with ouabain (OUA; 3 nM) for the indicated periods to assess levels of p75NTR by Western blot. Plots represent mean (±SEM) protein optical density (normalized to actin) expressed as a percentage of control (CT) group (set on 100%), with N = 3 experiments using independent retinal cultures. Data were analyzed by two-tailed Student t-test.

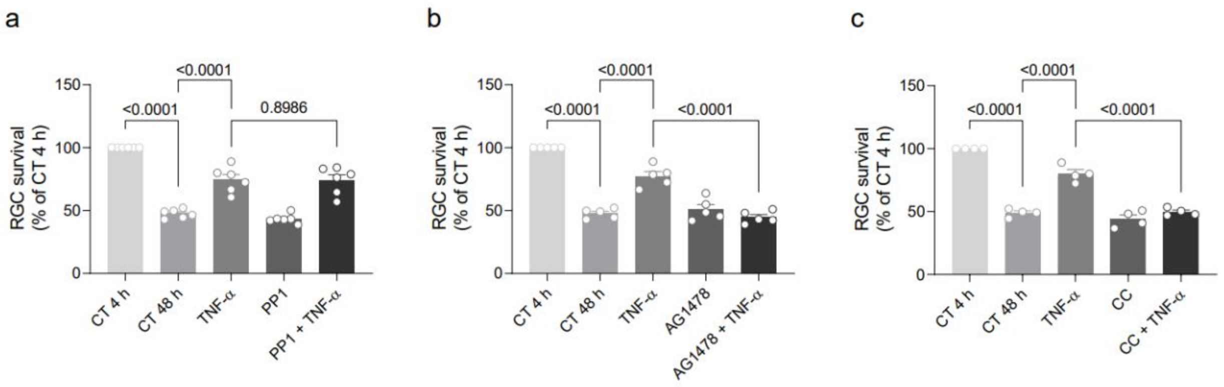

Figure S4. EGFR and PKC signaling pathways mediate TNF-α-induced RGC survival. Primary retinal cultures were treated with ouabain (OUA; 3nM) and/or the following drugs for 48 hours: (a) PP1 (Src inhibitor; 1 μM), (b) AG-1478 (EGFR inhibitor; 2.5 μM), or (c) chelerythrine chloride (CC; pan-PKC inhibitor; 1.25 μM). Plots represent mean (±SEM) RGC survival and are expressed as a percentage of control (CT) group at 4 hours in culture (set on 100%), with N = 4-6 experiments using independent retinal cultures. Data were analyzed by one-way ANOVA followed by Holm-Sidak's test.
